# Tea polyphenol protects against cisplatin-induced meiotic defects in porcine oocytes

**DOI:** 10.18632/aging.102084

**Published:** 2019-07-13

**Authors:** Changyin Zhou, Xue Zhang, Xiayan ShiYang, Huili Wang, Bo Xiong

**Affiliations:** 1 College of Animal Science and Technology, Nanjing Agricultural University, Nanjing 210095, China; 2 Institute of Animal Science, Jiangsu Academy of Agricultural Sciences, Nanjing 210014, China

**Keywords:** cisplatin, tea polyphenol, oocyte quality, meiotic failure, organelle dynamics

## Abstract

DDP (cisplatin), a DNA cross-linking agent, is one of the most common chemotherapeutic drugs that have been widely used in the treatment of sarcomas and germ cell tumors. DDP treatment exhibits severe side effects including renal toxicity, ototoxicity and embryo-toxicity. Women of reproductive age treated with DDP may lead to loss of primordial follicles, resulting in the depletion of the ovarian reserve and consequent premature ovarian failure. However, the influence of DDP on the oocyte quality and the strategy to prevent it has not yet fully clarified. Here, we report that DDP exposure resulted in the oocyte meiotic failure via disrupting the meiotic organelle dynamics and arrangement, exhibiting a prominently impaired cytoskeleton assembly, including spindle formation and actin polymerization. In addition, exposure to DDP led to the abnormal distribution of mitochondrion and cortical granules, two indicators of cytoplasmic maturation of oocytes. Conversely, TP (tea polyphenols) supplementation partially restored all of the meiotic defects resulted from DDP exposure through suppressing the increase of ROS level and the occurrence of DNA damage as well as apoptosis.

## INTRODUCTION

Cisplatin (DDP) was first described by Michele Peyrone in 1845, and its structure was determined in 1893 [1, 2]. After a long time of investigation, Rosenberg found its potential role in inducing the death of tumor cells and finally in 1978 it was approved by the FDA as a drug for the treatment of testicular and ovarian cancer [2]. Nowadays, DDP has been widely applied to treat a couple of tumors, such as ovary, testes, gastric, head and neck, non-small cell lung, gallbladder and urinary bladder [3–5]. DDP is an alkylating agent capable of forming adducts with macromolecules, particularly with N7 atoms of purine nucleobases, which results in inter- and intra-strand DNA cross-links [6, 7]. The inability to repair the DNA damage ultimately leads to the programed cell death [8]. In addition, experimental evidence has revealed that other mechanisms, such as the production of reactive oxygen species (ROS) and the activation of inflammatory pathways, may also contribute to the DDP-induced apoptosis [9].

Nevertheless, DDP treatment displays a variety of side effects including immunosuppression, renal toxicity, gastrointestinal disorders and ototoxicity [10]. It also causes gonadal suppression resulting in amenorrhea or azoospermia, partial or irreversible infertility and embryo-toxicity [11]. Additionally, as a first-line agent for ovarian cancer treatment [5, 12], DDP-induced gonadotoxicity leads to the changes in the menstrual cycle and loss of primordial follicles by apoptosis, resulting in the depletion of the ovarian reserve and consequent premature ovarian failure [13, 14]. However, the molecular mechanisms regarding how DDP influences the oocyte quality has not yet determined.

Tea, a popular beverage consumed world-wide, has been studied for its preventive effects against cancer as well as cardiovascular, neurodegenerative, and other diseases [15]. Most of the proposed beneficial effects in tea have been attributed to the polyphenolic compounds. TP (tea polyphenol) is known to be a strong antioxidant that can prevent the oxidative stress and DNA damage to modulate the carcinogen metabolism in many cell lines and animal models [15–20]. Furthermore, it has been reported that TP protects spermatogenesis against lonizing radiation-induced testicular injury in mice [21], improves the follicular development in rats, and promotes oocyte maturation as well as embryo development in bovines [22–25].

In current study, we used porcine oocytes as a research model to investigate the effects of DDP exposure on the oocyte quality because porcine oocytes and human oocytes have developmental and physiological similarities [26, 27]. We find that DDP exposure results in the meiotic failure in porcine oocytes through compromising the cytoskeleton organization, mitochondrial integrity and cortical granule distribution. Conversely, TP effectively suppresses the increased oxidative stress-induced DNA damage and apoptosis, thereby restoring the meiotic failure induced by DDP in oocytes.

## RESULTS

### TP alleviates the meiotic failure of oocytes exposed to DDP

**Figure 1:**
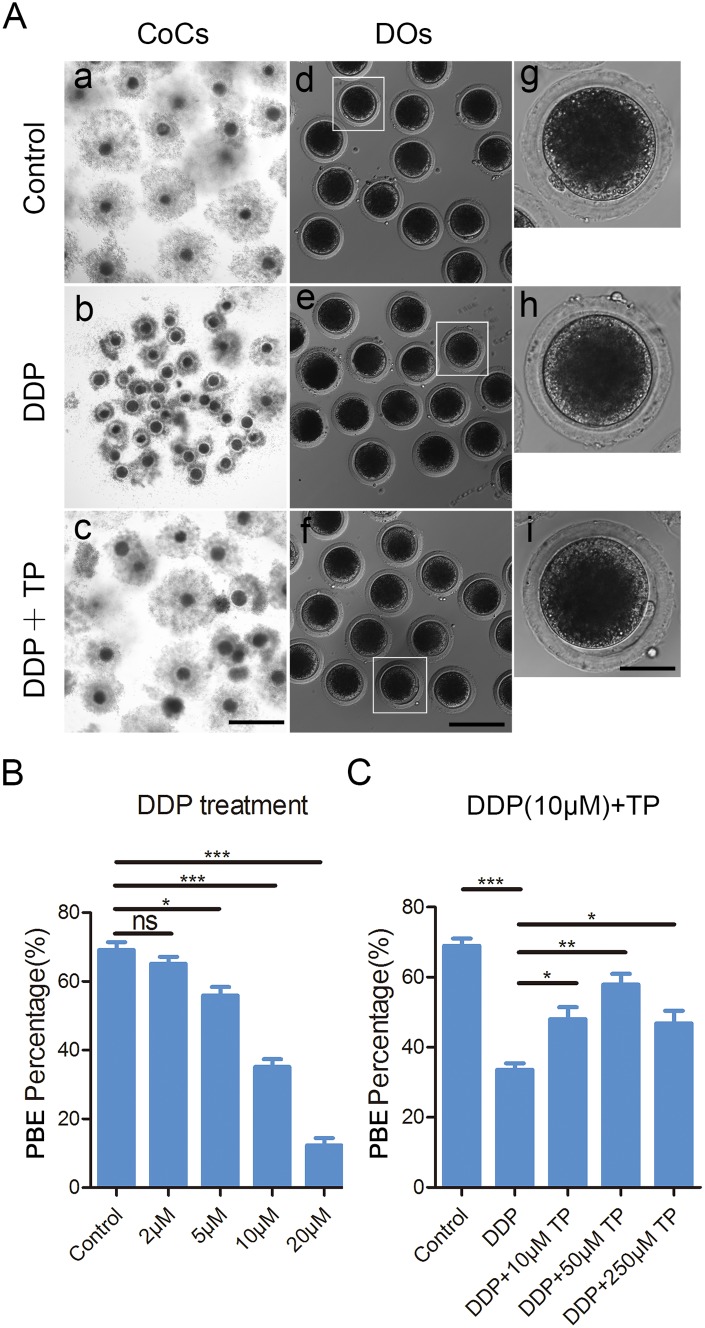
**Effects of different concentrations of DDP and TP on the porcine oocyte maturation.** (**A**) Representative images of oocyte meiotic progression in control, DDP -exposed and TP-supplemented oocytes. Cumulus cell expansion of COCs and polar body extrusion of DOs were imaged by the confocal microscope. Scale bar, 350 μm (a-c); 120 μm (d-f); 50 μm (g-i). (**B**) The rates of polar body extrusion were recorded in control and different concentrations of DDP-exposed groups (2 μM, 5 μM, 10 μM and 20 μM) after culture for 44 h *in vitro*. (**C**) The rates of polar body extrusion were recorded in control and different concentrations of TP -supplemented groups (10 μM, 50 μM and 250μM) after culture for 44 h with 10 μM DDP *in vitro*. Data in were presented as mean percentage (mean ± SEM) of at least three independent experiments. *P < 0.05, **P < 0.01, ***P < 0.001.

To examine the effects of DDP exposure on porcine oocyte meiotic maturation, different concentrations of DDP were supplemented to IVM medium (2, 5, 10 or 20 μM). After culturing for 44 h *in vitro*, we observed the developmental status of COCs and the proportion of polar body extrusion (PBE). As shown in Figure 1A, most of the cumulus cells surrounding COCs in the control group were fully expanded, while those in the DDP-exposed group were only partially expanded or not expanded (Figure 1A). Moreover, a majority of oocytes extruded the first polar body in the control group, but failed to do so after DDP treatment (Figure 1A). Quantitative data revealed that the proportion of PBE was reduced in a dose-dependent manner after DDP treatment compared with the controls (control: 69.2 ± 2.2%, n=149; 2 μM DDP: 65.2 ± 2.0%, n=144, P > 0.05; 5 μM DDP: 55.9 ± 2.4%, n=139, P < 0.05; 10 μM DDP: 35.1 ± 2.3%, n=149, P < 0.001; 20 μM DDP: 12.3 ± 2.2%, n=146, P < 0.001; Figure 1B). We selected the concentration of 10 μM DDP for further studies because it allowed a portion of oocytes to be matured for other studies.

To ask whether supplementation of TP would have beneficial impact on the meiotic defects caused by the DDP exposure, different concentrations of TP (10 μM, 50 μM and 250 μM) were supplemented into the maturation medium containing 10 μM DDP. The results showed that supplementation of 50 μM TP had the best improvement on the expansion of cumulus cells surrounding COCs and significantly increased the percentage of PBE compared with the DDP-exposed group (57.8 ± 3.4%, n=144 VS 34.5 ± 1.9%, n=140, P < 0.01; Figure 1A, 1C). Taken together, these observations suggest that the defects of porcine oocyte maturation induced by DDP exposure could be, at least partially, restored by the supplementation of TP.

### TP recovers the defects of spindle/chromosome structure in DDP-exposed oocytes

**Figure 2:**
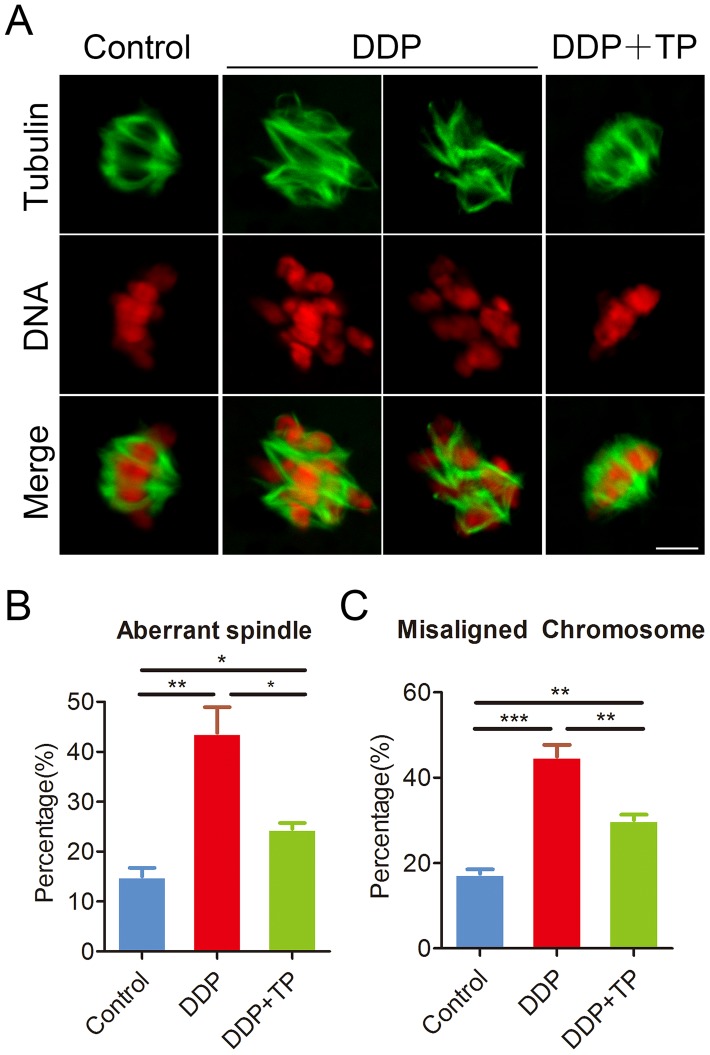
**Effects of TP on the spindle/chromosome defects in DDP-exposed porcine oocytes.** (**A**) Representative images of spindle morphologies and chromosome alignment in control, DDP-exposed and TP-supplemented oocytes. Scale bar, 5 μm. (**B**) The rate of aberrant spindles was recorded in control, DDP-exposed and TP-supplemented oocytes. (**C**) The rate of misaligned chromosomes was recorded in control, DDP-exposed and TP-supplemented oocytes. Data in were presented as mean percentage (mean ± SEM) of at least three independent experiments. *P < 0.05, **P < 0.01, ***P < 0.001.

Given that the arrest of oocyte meiotic progression is usually linked with the impairment of cytoskeleton structures [28, 29], we confirmed this possibility by staining the spindle structure and chromosome alignment in DDP-exposed oocytes. In control oocytes a standard barrel-shape spindle structure with a well-aligned chromosome on the equatorial plate was exhibited (Figure 2A). By contrast, a higher frequency of aberrant spindle morphologies with misaligned chromosomes was present in DDP-exposed oocytes (spindle: 15.0 ± 1.8%, n=114 VS 43.8 ± 5.1%, n=124, P < 0.01; chromosome: 17.5 ± 1.1%, n=109 VS 44.9 ± 2.8%, n=122, P < 0.001; Figure 2A–2C). Conversely, supplementation of TP in the DDP-exposed group substantially reduced the incidence of abnormal spindle/chromosome structure (spindle: 24.5 ± 1.2%, n=106, P < 0.05; chromosome: 30.1 ± 1.3%, n=120, P < 0.01; Figure 2A–2C).

### TP maintains the acetylation level of α-tubulin in oocytes exposed to DDP

**Figure 3:**
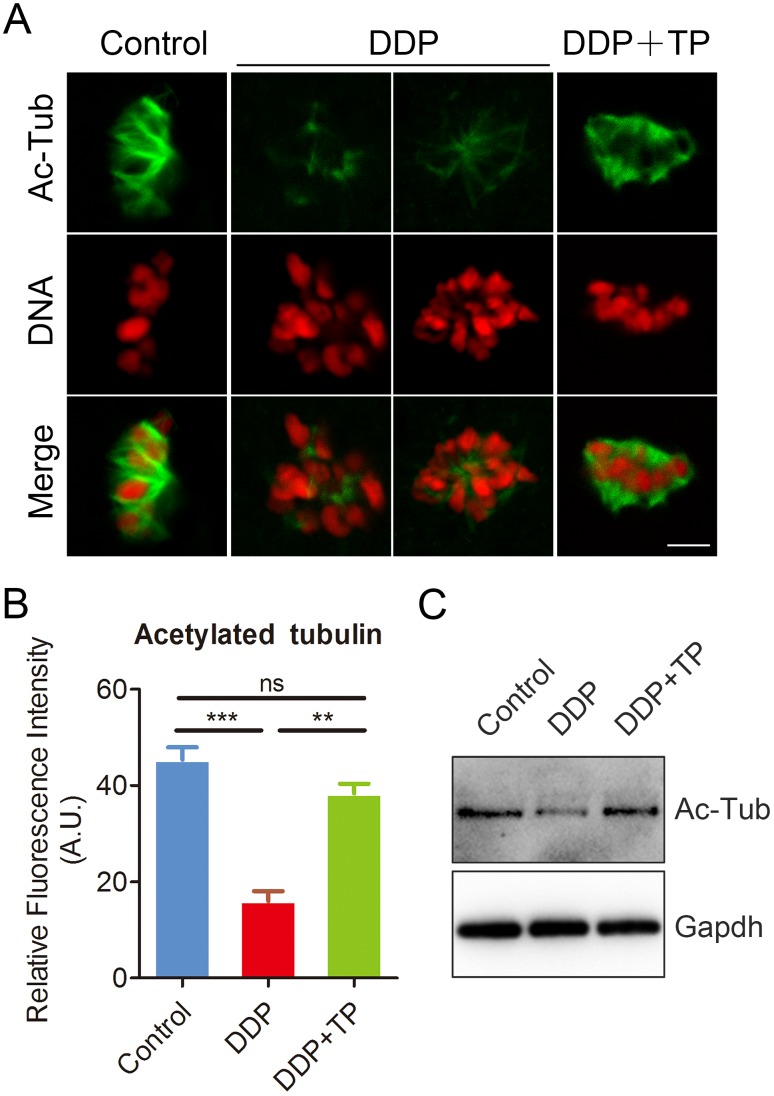
**Effects of TP on the acetylation level of α-tubulin in DDP-exposed porcine oocytes.** (**A**) Representative images of acetylated α-tubulin in control, DDP-exposed and TP-supplemented oocytes. Scale bar, 5 μm. (**B**) Quantitative analysis of the fluorescence intensity of acetylated α-tubulin in control, DDP-exposed and TP-supplemented oocytes. (**C**) The acetylation levels of α-tubulin were detected by Immunoblotting in control, DDP-exposed and TP-supplemented oocytes. The blots were probed with anti-acetyl-α-tubulin and anti-Gapdh antibodies, respectively. Data were presented as mean percentage (mean ± SEM) of at least three independent experiments. **P < 0.01, ***P < 0.001.

Studies have shown that the disorganized spindle structure might be resulted from the disruption of acetylation level of α-tubulin, an indicator of stable microtubules in oocytes [30–33]. Therefore, we next assessed the level of acetylated α-tubulin in oocytes exposed to DDP. Immunostaining analysis and quantitative measurement of the fluorescence intensity indicated that α-tubulin in DDP-exposed oocytes displayed an apparently decreased level of acetylation compared with the controls (16.0 ± 2.1, n=52 VS 45.4 ± 2.6, n=55, P < 0.001; Figure 3A, 3B). While TP supplementation remarkably raised the signals of acetylated α-tubulin in DDP-exposed oocytes (38.3 ± 2.0, n=57 VS 16.0 ± 2.1, n=52, P < 0.01). These observations were further confirmed by the immunoblotting analysis (Figure 3C), implying that the recovery of perturbed spindle structure in DDP-exposed oocytes by TP supplementation might be mediated by the maintenance of acetylation level of α-tubulin.

### TP restores the polymerization of actin filaments in DDP-exposed oocytes

**Figure 4:**
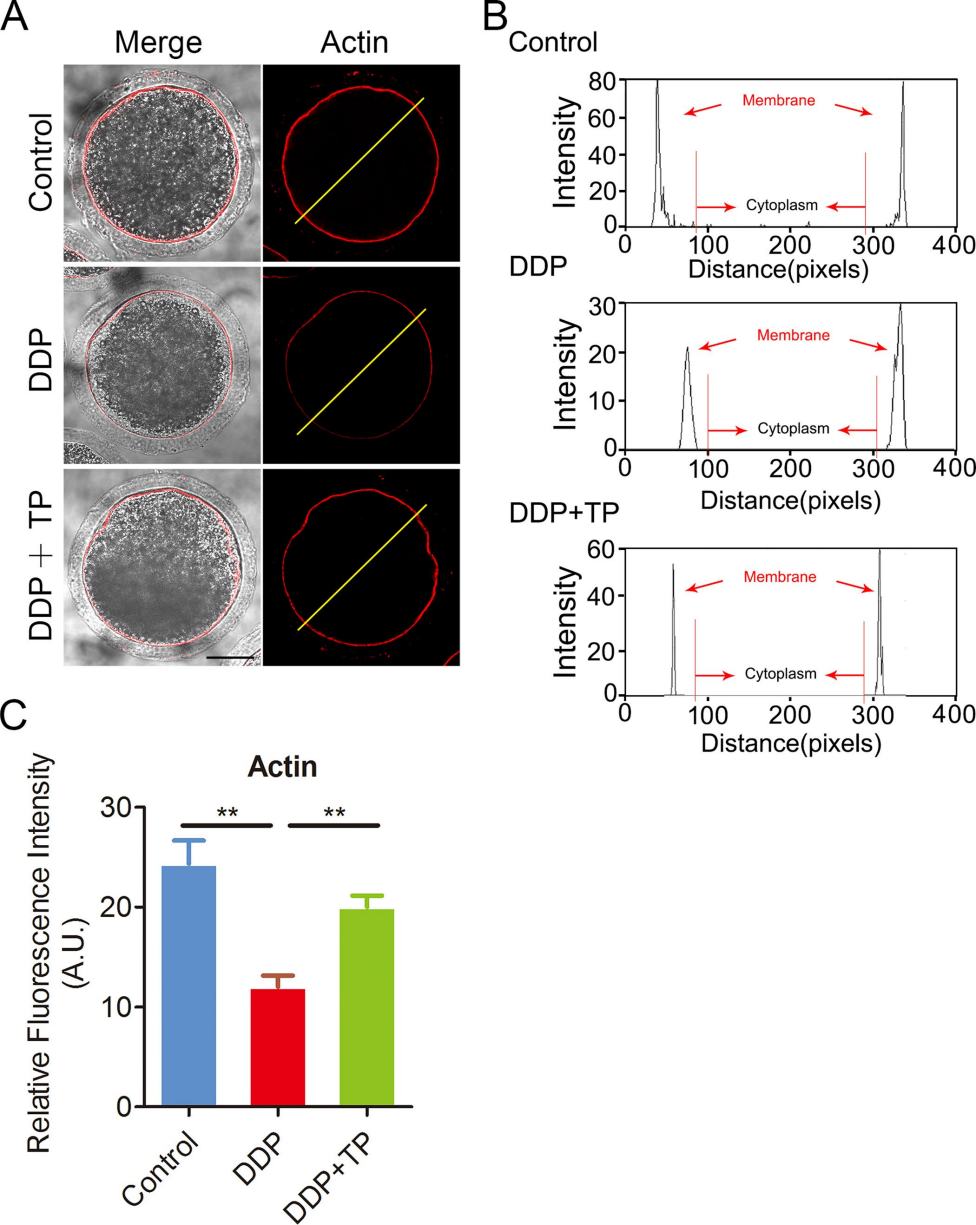
**Effects of TP on the actin polymerization in DDP-exposed porcine oocytes.** (**A**) Representative images of actin filaments in control, DDP-exposed and TP-supplemented oocytes. Scale bar, 30 μm. (**B**) Right graphs show fluorescence intensity profiling of phalloidin in oocytes. Lines were drawn through the oocytes, and pixel intensities were quantified along the lines. (**C**) The fluorescence intensity of actin signals was measured in control, DDP-exposed and TP-supplemented oocytes. Data were presented as mean percentage (mean ± SEM) of at least three independent experiments. **P < 0.01.

During oocyte meiotic maturation, the actin assembly plays a critical role in asymmetric spindle positioning and cortical polarization [34–36]. To investigate whether the meiotic defects induced by DDP exposure involves the actin dynamics, phalloidin-TRITC was employed to stain the F-actin to observe the polymerization of actin filaments. As shown in Figure 4A, actin filaments were concentrated uniformly on the plasma membrane with robust signals in control oocytes. By contrast, DDP-exposed oocytes displayed the impaired assembly of actin filaments with weak signals (Figure 4A), which was confirmed by the fluorescence plot profiling that was quantified along the lines drawn through the oocytes (Figure 4B). In addition, the quantitative measurement of fluorescence intensity on the membrane revealed that actin signals were substantially decreased in DDP-exposed oocytes, but increased to the control comparable level after supplementation with TP (control: 24.4 ± 2.2, n=47, P < 0.01; DDP: 12.3 ± 1.1, n=49; TP: 22.3 ± 1.2, n=53, P < 0.01; Figure 4C), indicating that TP prevents the assembly of actin cytoskeleton from damage induced by DDP exposure.

### TP rescues the distribution of CGs and mitochondria in DDP-exposed oocytes

**Figure 5:**
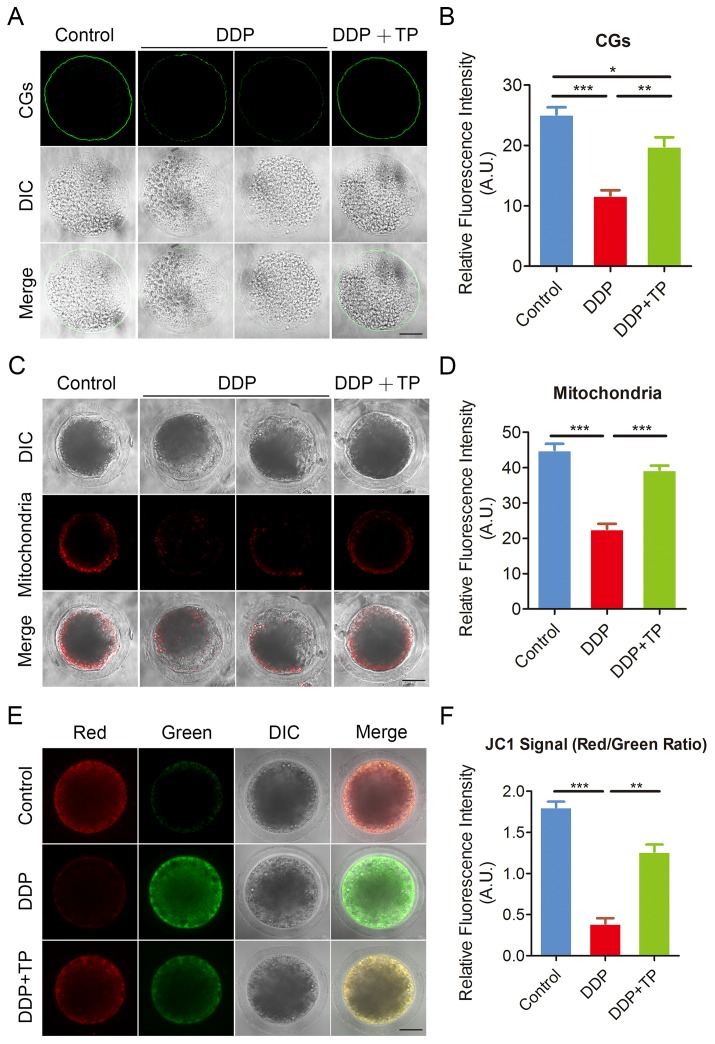
**Effects of TP on the distribution of CGs and mitochondria in DDP-exposed porcine oocytes.** (**A**) Representative images of CGs localization in control, DDP-exposed and TP-supplemented oocytes. Scale bar, 40 μm. (**B**) The fluorescence intensity of cortical granules was measured in control, DDP-exposed and TP-supplemented oocytes. (**C**) Representative images of mitochondria in control, DDP-exposed and TP-supplemented oocytes. Scale bar, 35 μm. (**D**) The immunofluorescence intensity of mitochondrion signals was recorded in control, DDP-exposed and TP-supplemented oocytes. (**E**) Mitochondrial membrane potential (ΔΨm) was detected by JC-1 in control, DDP-exposed and TP-supplemented oocytes (Red, high ΔΨm; Green, low ΔΨm). (**F**) The ratio of red and green fluorescence intensity was recorded in control, DDP-exposed and TP-supplemented oocytes. Data were presented as mean percentage (mean ± SEM) of at least three independent experiments. *P < 0.05, **P < 0.01, ***P < 0.001.

The distribution of CGs (cortical granules) and mitochondria are two key indexes of cytoplasmic maturation of oocytes [37, 38]. CGs are triggered at fertilization to migrate to the plasma membrane of oocytes, where they fuse and exocytose their contents to prevent polyspermy [39]. The results from Figure 5A showed that the signals of CGs exhibited markedly faded following culture with DDP. The quantification analysis also confirmed that the fluorescence intensity of the signals had a significant decline in DDP-exposed oocytes compared to the controls (11.7 ± 0.9, n=52 VS 25.1 ± 1.2, n=51, P < 0.001; Figure 5B), and this decline was rescued by the supplementation of TP during *in vitro* culture (19.9 ± 1.5, n=47, P < 0.01; Figure 5B).

In normal porcine oocytes most of mitochondria accumulated around lipid droplets in the subcortical region, but lost the specific localization in DDP-exposed oocytes (Figure 5C). The measurement of fluorescence intensity showed that signals of mitochondria prominently reduced in the DDP-exposed oocytes in comparison with the controls (22.6 ± 1.5, n=58 VS 45.1 ± 1.8, n=55, P < 0.001; Figure 5C, 5D). Conversely, this defect prevented by the treatment with TP (39.3 ± 1.2, n=49, P < 0.001; Figure 5C, 5D). We also assessed the mitochondrial membrane potential (ΔΨm) by JC-1 staining. Mitochondria with high membrane potential showed a red fluorescence while those with low membrane potential showed a green fluorescence (Figure 5E). The quantitative analysis revealed that the ratio of red and green fluorescence was significantly higher in control oocytes than that in DDP-exposed oocytes, but rescued in TP-supplemented oocytes (1.8 ± 0.07, n=27, P < 0.001 VS 0.4 ± 0.07, n=28 VS 1.3 ± 0.09, n=27, P < 0.01; Figure 5F). Altogether, these observations indicate that TP restores the impairment of cytoplasmic maturation of oocytes exposed to DDP.

### TP suppresses oxidative stress, DNA damage and apoptosis induced by DDP exposure in porcine oocytes

**Figure 6:**
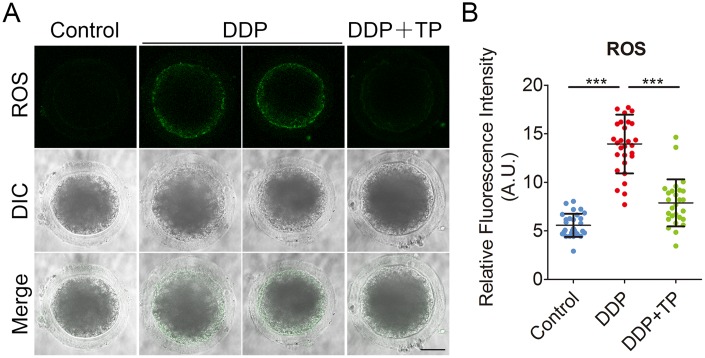
**Effects of TP on the ROS level in DDP-exposed porcine oocytes.** (**A**) Representative images of ROS levels in control, DDP-exposed and TP-supplemented oocytes. Scale bar, 35 μm (**B**) The fluorescence intensity of ROS in control, DDP-exposed and TP-supplemented oocytes were measured by the confocal microscopy using identical settings and parameters. Data were presented as mean percentage (mean ± SD) of at least three independent experiments. ***P < 0.001.

DDP is known to induce oxidative stress in various cells to impair the cellular functions [40, 41], we then tested if the defects observed in DDP-exposed porcine oocytes were mediated by this mechanism. To verify this assumption, we compared the ROS levels between control and DDP-exposed oocytes by DCFH staining. The results showed that in control oocytes the signals were hardly detected (Figure 6A). However, DDP exposure remarkably increased the green signals in the cytoplasm of oocytes (Figure 6A). Consistently, the fluorescent intensity of ROS was significantly increased in DDP-exposed oocytes compared to controls (14.0 ± 0.6, n=29 VS 5.6 ± 0.2, n=29, P < 0.001; Figure 6B). Supplementation with TP effectively reduced the levels of ROS in DDP-exposed oocytes (7.9 ± 0.5, n=27, P < 0.001; Figure 6A, 6B).

**Figure 7:**
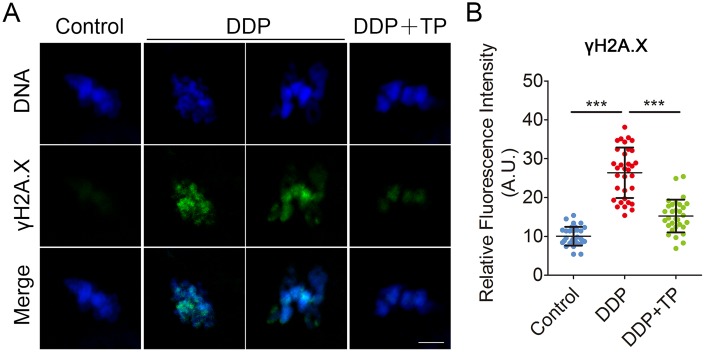
**Effects of TP on DNA damage in DDP-exposed porcine oocytes.** (**A**) Representative images of DNA damage in control, DDP-exposed and TP-supplemented groups. Scale bar, 5 μm. (**B**) The fluorescence intensity of γH2A.X signals was measured in control, DDP-exposed and TP-supplemented groups. Data in (**B**) were presented as mean percentage (mean ± SD) of at least three independent experiments. ***P < 0.001.

**Figure 8:**
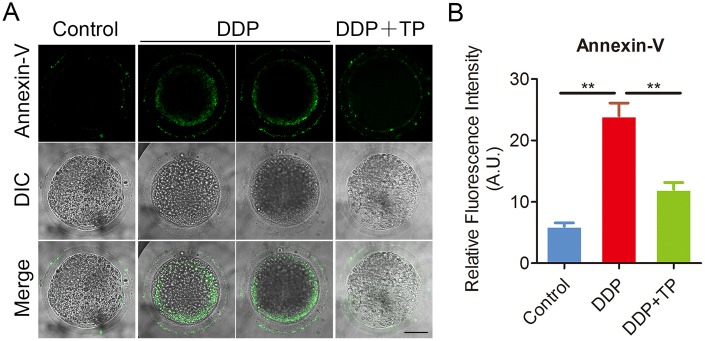
**Effects of TP on early apoptosis in DDP-exposed porcine oocytes.** (**A**) Representative images of apoptotic oocytes in control, DDP-exposed and TP -supplemented groups. Scale bar, 35 μm. (**B**) The rate of early apoptosis was recorded in control, DDP-exposed and TP-supplemented oocytes. Data in (**B**) were presented as mean percentage (mean ± SEM) of at least three independent experiments. **P < 0.01.

Since the excessive oxidative stress always induces DNA damage and apoptosis [42, 43], we next assessed DNA damage and the early apoptosis in oocytes by γ-H2A.X and Annexin-V staining. The immunofluorescence results showed that the foci of γ-H2A.X were accumulated on the DNA in DDP-exposed oocytes instead of control oocytes, in agreement with the quantification data showing that the intensity of γ-H2A.X signals was remarkably elevated in DDP-exposed oocytes in comparison with the controls (26.4 ± 1.1, n=33 VS 10.1 ± 0.4, n=32, P < 0.001; Figure 7A, 7B). While treatment with TP markedly decreased the occurrence of DNA damage (15.2 ± 0.8, n=31, P < 0.001; Figure7A, 7B). Also, we observed that the fluorescent signals of Annexin-V were rarely present in control oocytes, but clearly found on the plasma membrane of DDP-exposed oocytes (Figure 8A). Concordantly, the rate of apoptotic oocytes was dramatically higher in DDP-exposed group than that in controls (24.0 ± 2.0, n=36 VS 6.5 ± 0.7, n=38, P < 0.01; Figure 8A, 8B), but rescued in TP -supplemented group (11.8 ± 0.9, n=41; P < 0.01; Figure 8A, 8B).

## DISCUSSION

DDP, one of the most common chemotherapeutic drugs, is a DNA cross-linking agent widely used in the treatment of sarcomas and germ cell tumors. Its precise mechanism of action is not entirely clear, although it is known to intercalate with DNA strands causing crosslinking and adduct formation. DDP is categorized as a member of the intermediate gonadal risk group of drugs and has been reported to cause ovarian failure [6, 44]. However, the effects of DDP exposure on the oocyte quality have not yet defined.

To address this question, we investigated the critical biological events occurred during meiotic progression that drives the nuclear and cytoplasmic maturation of oocytes. Our findings revealed that oocytes exhibited a remarkably reduced proportion of polar body extrusion with impaired expansion of cumulus cells when treated with increasing concentrations of DDP, suggesting that DDP exposure perturbs the normal oocyte meiotic maturation. Further, the compromised nuclear maturation of oocytes was evidenced by the observations that spindle structure and chromosome alignment were severely disrupted in DDP-exposed oocytes. The abnormalities of spindle assembly prompted us to assess the microtubule dynamics by evaluating the acetylation level of tubulin, a posttranslational modification that occurs on Lys-40 of the α-tubulin subunit to stabilize the microtubules in oocytes [32, 45]. Expectedly, our data illustrated that DDP exposure decreased the acetylation level of α-tubulin, indicating that the loss of microtubule stability might be one of the major causes leading to the disorganized spindle structure. Additionally, the dynamics of actin filament, another indispensable component of cytoskeleton, has been reported to play important roles in spindle positioning and meiotic progression during oocyte meiosis [46–48]. Our findings validated that the perturbed actin polymerization might be another major reason resulting in the oocyte meiotic failure when exposed to DDP.

Mitochondrion is a primary organelle that provides the source of ATP for oocyte development [49–51], and its integrity has been regarded as one of the key indexes of cytoplasmic maturation of oocytes. Our data found that the distribution of mitochondria was considerably impaired in oocytes exposed to DDP, suggesting that DDP exposure compromises the cytoplasmic maturation of oocytes via damaging the functionality of the mitochondria. Furthermore, another sign of oocyte cytoplasmic maturation is the proper distribution of cortical granules, a type of oocyte-specific vesicles that locate in the subcortical region of oocytes to exert functions in the prevention of polyspermy following fertilization [39]. We showed that the amount of cortical granules in the subcortex of DDP-exposed oocytes were significantly reduced, confirming the defective cytoplasmic maturation of oocytes as a result of DDP exposure.

Finally, we found that DDP-exposed porcine oocytes displayed a much higher level of ROS and the incidence of DNA damage as well as apoptosis, which might be cause for the DDP exposure-induced low quality of oocytes. More importantly, we documented that TP supplementation is able to, at least partially, recover all of the meiotic defects we observed in DDP-exposed oocytes via eliminating the excessive ROS, which in turn inhibits the accumulation of DNA damage and occurrence of apoptosis. These findings potentially provide an effective strategy to ameliorate the quality of oocytes from DDP-treated patients with cancer.

## MATERIALS AND METHODS

### Reagents

Mouse monoclonal anti-α-tubulin FITC antibody, anti-phalloidin-TRITC antibody, anti-acetyl-α-tubulin (Lys-40) antibody and lens culinaris agglutinin (LCA)-FITC were purchased from Sigma (St. Louis, MO, USA); FITC-conjugated goat anti-mouse IgG (H + L) and TRITC-conjugated goat anti-mouse IgG (H + L) were purchased from Zhongshan Golden Bridge Biotechnology Co., LTD (Beijing, China). DDP was purchased from Beijing Solarbio Science and Technology Co., LTD. (Beijing, China). TP was from Selleck Chemicals (Houston, TX, USA).

### Oocyte collection and *in vitro* maturation (IVM)

For *in vitro* maturation (IVM), ovaries were collected from prepubertal gilts at a local abattoir and transported to the laboratory in a 0.9% NaCl solution containing penicillin G (75 mg/ml) and streptomycin sulphate (50 mg/ml). Soon afterwards ovaries were washed twice with sterile phosphate-buffered saline (PBS), the Cumulus-oocyte complexes (COCs) were subsequently aspirated from medium-sized follicles (3–6 mm in diameter) using a 20-gauge needle attached to a 20 ml disposable syringe. COCs surrounded by a compact cumulus mass with evenly granulated cytoplasm were washed three times with maturation medium, separated from the cellular debris, and then transferred to the maturation medium. The basic maturation medium was improved TCM-199 supplemented with 75 μg/ml of penicillin, 50 μg/ml of streptomycin, 0.5 μg/ml of LH, 0.5 μg/ml of FSH, 10 ng/ml of EGF and 0.57 mM cysteine. To prepare mature oocytes *in vitro*, a group of 30 COCs was transferred to 100 μl of maturation medium and then covered with 100 μl paraffin oil to culture at 38.5°C in a humidified atmosphere of 5% CO_2_.

### DDP and TP treatment

DDP was dissolved in DMF then in TCM-199 culture medium to a final concentration of 2,5,10 and 20 μM. TP was dissolved in PBS and diluted with maturation medium to a final concentration of 10 μM, 50 μM and 250 μM. It should be added to maturation medium immediately before use. The final concentrations of DMF in the culture medium were not more than 0. 1%.

### Immunofluorescence staining and confocal microscopy

DOs were collected and fixed in 4% paraformaldehyde (PFA) in PBS for 30 min at room temperature. They were then washed three times in wash buffer, and were permeabilized in 1% Triton X-100 (in PBS) for 1 h at room temperature. After three further washes, they were blocked with blocking buffer (1% BSA-supplemented PBS) for 1 h at room temperature to suppress the non-specific binding of IgG. DOs were then incubated with primary antibodies overnight at 4°C, washed three more times, and incubated with secondary antibody for 1 h at room temperature. Finally, propidium iodide (PI) was used to stain nuclei for 10 min at room temperature. Samples were mounted on glass slides and examined with a confocal laser-scanning microscope (LSM 700 META; Zeiss, Germany).

### Immunoblotting analysis

A total of 120 porcine oocytes was collected and lysed in 4× NuPAGE™ LDS sample buffer (ThermoFisher, USA) containing protease inhibitor, and then separated on 10% Bis-Tris precast gels and transferred onto polyvinylidene difluoride (PVDF) membranes. The blots were blocked in Tris buffered saline Tween 20 (TBST) containing 5% low fat dry milk for 1 h at room temperature and then incubated with anti-acetylated α-tubulin antibody (1:1000) or anti-Gapdh (1:5000) antibody overnight at 4°C. After washing in TBST, the blots were incubated with horseradish peroxidase (HRP)-conjugated secondary antibodies for 1 h at room temperature. Chemiluminescence was detected with ECL Plus (GE Healthcare, USA) and protein bands were visualized by Tanon-3900 (Tanon, China).

### Evaluation of mitochondrion distribution

Matured oocytes were stained for active mitochondria in the maturation medium containing 500 nM cell permeant MitoTracker Red CMXRos (ThermoFisher) for 30 min at 38.5°C in a dark environment and 5% CO2 in air. After washing three times with maturation medium, oocytes were mounted on non-fluorescent glass slides and observed under the laser-scanning confocal microscope.

### Assessment of mitochondrial membrane potential

Mitochondrial membrane potential was evaluated using MitoProbe JC-1 Assay Kit (M34152, Thermo, USA). Briefly, oocytes were cultured in TCM-199 medium with 2 μM JC-1 for 30 min at 38.5°C, followed by washing with buffer for 10 min. Samples were immediately imaged in a glassbottom dish on the laser scanning confocal microscope. JC-1 dye exhibits potential-dependent accumulation in mitochondria, indicated by a fluorescence emission shift from green (~529 nm) to red (~590 nm). Thus, mitochondrial depolarization is indicated by a decrease in the red/green fluorescence intensity ratio. ImageJ software was used to quantify the intensity of fluorescence.

### Determination of ROS generation

To determine the levels of intracellular ROS production, denuded oocytes were incubated with the oxidation-sensitive florescent probe dichloroflorescein (DCFH) for 30 min at 38.5°C in D-PBS that contained 10 μM DCFH diacetate (DCFHDA) (Beyotime Institute of Biotechnology, China). Then oocytes were washed three times in D-PBS containing 0.1% BSA, placed on glass slides and observed under the confocal microscope.

### Annexin-V staining

Denuded oocytes were stained with the Annexin-V staining kit (Vazyme, Nanjing, China) according to the manufacturer’s instruction. After washing twice in PBS, the viable oocytes were stained for 30 min in the dark with 90μl of binding buffer containing 10μl of Annexin-V-FITC. Then oocytes were washed three times in D-PBS containing 0.1% BSA, placed on glass slides and observed under the confocal microscope.

### Statistical analysis

All percentages from at least three repeated experiments were expressed as mean ± SEM or mean ± SD, and the number of oocytes observed was labeled in parenthesesas (n). Data were analyzed by one-way ANOVA or *t* test, which was provided by SPSS 16.0 statistical software (SPSS, Chicago, IL, USA). The level of significance was accepted as *P < 0.05*.
